# Novel gene rearrangement in the mitochondrial genome of *Anastatus fulloi* (Hymenoptera Chalcidoidea) and phylogenetic implications for Chalcidoidea

**DOI:** 10.1038/s41598-022-05419-0

**Published:** 2022-01-25

**Authors:** Jiequn Yi, Han Wu, Jianbai Liu, Jihu Li, Yinglin Lu, Yifei Zhang, Yinjie Cheng, Yi Guo, Dunsong Li, Yuxing An

**Affiliations:** 1 Institute of Nanfan & Seed Industry, Guangdong Academy of Sciences, Guangzhou, 510316 China; 2 Guangdong Provincial Key Laboratory of High Technology for Plant Protection/Plant Protection Research Institute, Guangdong Academy of Agricultural Sciences, Guangzhou, 510640 China

**Keywords:** Evolutionary biology, Phylogenetics

## Abstract

The genus *Anastatus* comprises a large group of parasitoids, including several biological control agents in agricultural and forest systems. The taxonomy and phylogeny of these species remain controversial. In this study, the mitogenome of *A. fulloi* Sheng and Wang was sequenced and characterized. The nearly full-length mitogenome of *A. fulloi* was 15,692 bp, compromising 13 protein-coding genes (PCGs), 2 rRNA genes, 22 tRNA genes and a control region (CR). The total A + T contents were 83.83%, 82.18%, 87.58%, 87.27%, and 82.13% in the whole mitogenome, 13 PCGs, 22 tRNA genes, 2 rRNA genes, and CR, respectively. The mitogenome presented negative AT skews and positive GC skews, except for the CR. Most PCGs were encoded on the heavy strand, started with ATN codons, and ended with TAA codons. Among the 3736 amino acid-encoding codons, TTA (Leu1), CGA (Arg), TCA (Ser2), and TCT (Ser2) were predominant. Most tRNAs had cloverleaf secondary structures, except trnS1, with the absence of a dihydrouridine (DHU) arm. Compared with mitogenomes of the ancestral insect and another parasitoid within Eupelmidae, large-scale rearrangements were found in the mitogenome of *A. fulloi*, especially inversions and inverse transpositions of tRNA genes. The gene arrangements of parasitoid mitogenomes within Chalcidoidea were variable. A novel gene arrangement was presented in the mitogenome of *A. fulloi*. Phylogenetic analyses based on the 13 protein-coding genes of 20 parasitoids indicated that the phylogenetic relationship of 6 superfamilies could be presented as Mymaridae + (Eupelmidae + (Encyrtidae + (Trichogrammatidae + (Pteromalidae + Eulophidae)))). This study presents the first mitogenome of the *Anastatus* genus and offers insights into the identification, taxonomy, and phylogeny of these parasitoids.

## Introduction

Mitochondria are double-membrane-bound organelles that are widely found in eukaryotic cells^1,2^. Their genomes, i.e., mitogenomes, are small in size and have the characteristics of maternal inheritance, conserved gene components, absence of introns, rare recombination, and a relatively high evolution rate^3–5^. Therefore, the mitogenome is a suitable molecular marker that has been widely applied in molecular identification, evolution, and phylogeny^6–8^. In recent years, an increasing number of mitogenomes have been sequenced, analysed, and deposited in the NCBI database^9–11^. These mitogenomes provide valuable information not only about nucleotide composition but also genome-level characteristics^12^.

In general, a typical mitogenome of an insect is a circular molecule with double strands, ranging from 14 to 19 kb in size^13,14^. It usually contains four components, including 13 protein-coding genes (PCGs), 2 ribosomal RNA genes (rRNAs), 22 transfer RNA genes (tRNAs), and a control region (CR)^15,16^. Most insect mitogenomes share an identical gene order; however, rearrangements of genes have been found in the mitogenomes of some species, and these rearrangements include transposition, inversion, inverse transposition, and tandem duplication random loss (TDRL)^5,17^. To date, the base composition and gene order of mitogenomes have been extensively applied to clarify evolutionary events in certain groups of insects^18–20^.

Most *Anastatus* (Hymenoptera: Chalcidoidea) species are parasitoids of numerous insect species, and several species of *Anastatus* have been evaluated as biological control agents for various pests in agricultural and forest systems^21,22^. These insects represent a large family with approximately 150 recognized species but remain largely unstudied^23^. The current morphology-based taxonomy of *Anastatus* is problematic due to early taxonomic confusion, low degrees of morphological differentiation, and morphological variation related to host and geographical origin^23–25^. Parasitoids within Chalcidoidea are some of the most diverse hymenopterous insects, exhibiting the characteristics of frequent gene rearrangement, high substitution rates, and a strong base composition bias in mitogenomes^26,27^. Although the mitogenomes of other hymenopterous insects have been applied for identification, evolution, and phylogeny, this is not the case for *Anastatus* parasitoids. To date, mitogenomes of *Anastatus* parasitoids are still not available in the NCBI database.

Next-generation sequencing (NGS) is an increasingly applied technology used to obtain large-scale genetic information. It provides the full-length sequences of insect mitogenomes practically and effectively. In the present study, we applied NGS to obtain the complete mitogenome of *A. fulloi* Sheng and Wang, which represented the first sequenced mitochondrial genome in the *Anastatus* genus. The mitogenome was characterized, including the structure of the mitogenome, gene organization, nucleotides, amino acid composition, codon usage, and tRNA secondary structure. Gene rearrangement was discussed between several mitogenomes of parasitoids and the ancestral insect. In addition, phylogenetic analyses were performed based on the 13 PCGs of 20 parasitoids within Chalcidoidea. This study provides information about the taxonomy and phylogeny of this special insect and unveils the phylogenetic position of *Anastatus* within Chalcidoidea.

## Methods

### Insect collection and DNA extraction

Adult specimens of *A. fulloi* were obtained from the Plant Protection Research Institute, Guangdong Academy of Agricultural Sciences, People’s Republic of China. All specimens were preserved in 100% alcohol and stored at − 20 °C. Total genomic DNA was extracted by using a DNeasy tissue kit (Qiagen, Hilden, Germany) according to the manufacturer’s protocols. Subsequently, total DNA was quantified and tested by a Qubit fluorometer with a dsDNA high-sensitivity kit (ThermoFisher, Foster City, CA, USA) and agarose gel (1%) electrophoresis.

### Mitochondrial genome sequencing and assembly

At least 1 μg of DNA was used to construct the sequencing library using the TruSeq DNA Library Preparation kit according to standard protocols. The library was then sequenced by the Illumina HiSeq™4000 (Illumina, USA) platform with paired-end reads of 2 × 150 bases. A total of 4.79 Gb of clean data was obtained with a Q30 of 92.16%. SPAdes v3.10.1 (http://bioinf.spbau.ru/spades)^28^ and A5-miseq v20150522^29^ were used to assemble the clean data into contigs and scaffolding. The sequences with high sequencing coverage were extracted to identify the mitochondrial sequences by the NCBI NT library using BLASTN (BLAST v2.2.31+). MUMmer v3.1^30^ was used to determine the contig positions and fill the gaps between contigs. Through Pilon v1.18^31^, the results were error-corrected to obtain the final mitochondrial sequence.

### Genome annotation and analysis

The mitogenome of *A. fulloi* was annotated on MITOS (http://mitos.bioinf.uni-leipzig.de/index.py). The secondary structure of tRNAs was also predicted by MITOS. Annotation results were confirmed by comparison with homologous sequences in the NCBI database, and then submitted to NCBI. A mitogenome map of *A. fulloi* was generated by mtviz (http://pacosy.informatik.uni-leipzig.de/mtviz/mtviz).

The base composition and relative synonymous codon usage (RSCU) values of PCGs were calculated by MEGA 6.0. The AT and GC skews were counted using the following formulas: AT skew = (A − T)/(A + T) and GC skew = (G − C)/(G + C). The gene orders of parasitoid mitogenomes within Chalcidoidea were compared by CREx (http://pacosy.informatik.uni-leipzig.de/crex/form#INFO)^32^.

All mitogenome information from 21 species was downloaded, including 19 parasitoids within Chalcidoidea and 2 outgroups. Together with the mitogenome of *A. fulloi*, they were used to extract the 13 PCGs through PhyloSuite v1.2.2^33^. These PCGs were aligned by MAFFT 7.149^34^ and MACSE v. 2.03^35^. Conserved blocks were obtained using Gblocks v0.91b^36^. MrBayes on XSEDE and RAxML on XSEDE (CIPRES portal) were employed to construct the bayesian inference (BI) and maximum likelihood (ML) phylogenetic trees, respectively. ModelFinder was used to evaluate the best evolutionary model^37^. GTR + F + I + G4 was selected by the Bayesian information criterion (BIC). The MrBayes analyses ran as 4 independent Markov chains for 3 million generations, sampled every 1000 generations. A burn-in of 25% was used to generate the consensus tree. The RAxML analysis was performed with 1000 replicates of ultrafast likelihood bootstrap. FigTree v1.4.2 was employed to edit two phylogenetic trees.

## Results and discussion

### General features of the mitogenome

**Figure 1:**
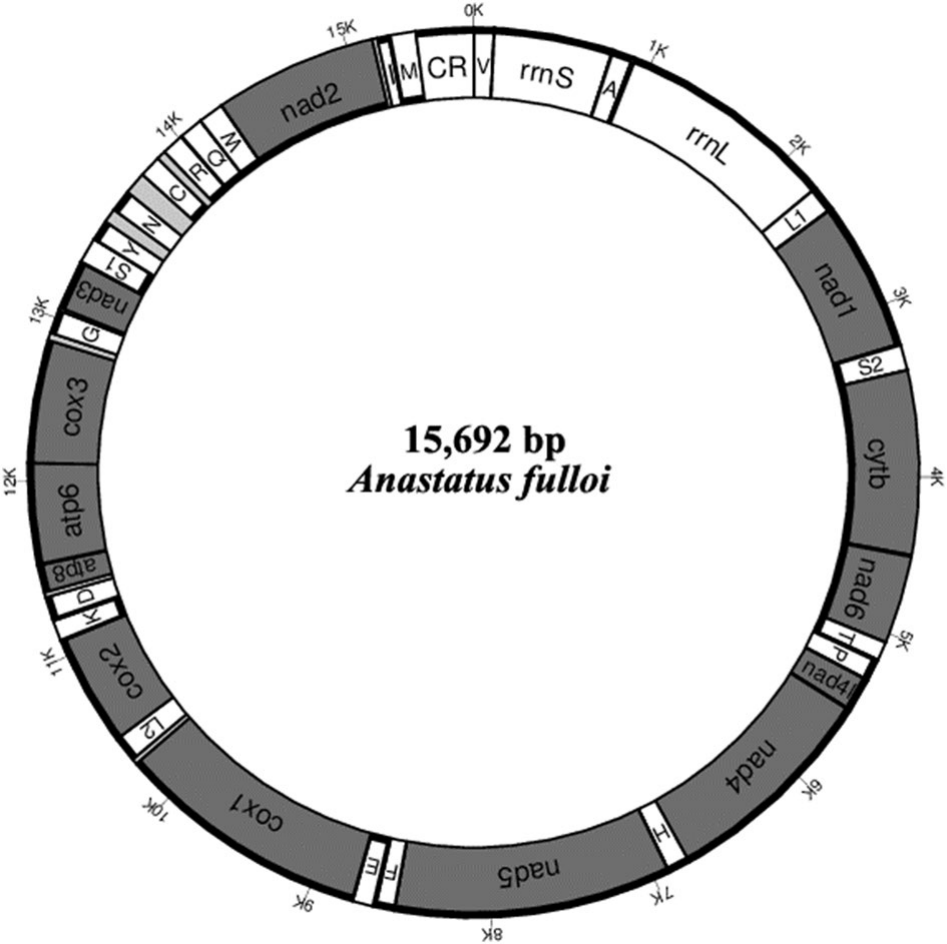
Mitochondrial map of *Anastatus fulloi*. PCGs are marked in grey. Genes marked with bold lines outside the circle map are encoded on the heavy strand.

The mitochondrial sequence of *A. fulloi* has been submitted to GenBank with the accession number OK545741. The nearly complete mitogenome was 15,692 bp in length, within the range of other hymenopterous mitogenomes: from 15,137 (*Idris sp.*) to 20,370 bp (*Trachelus iudaicus*)^38,39^. We failed to obtain the complete CR using next-generation and Sanger sequencing, which may be attributed to the comparatively high A + T content and presence of repeat units in CR^19,40,41^. Therefore, the *A. fulloi* mitogenome contained 37 genes (13 protein-coding genes, 2 rRNA genes, and 22 tRNA genes) and a partial CR (Table 1, Fig. 1). This is a common gene set for most hymenopterous mitogenomes^17,42,43^.

**Table 1:** Characteristics of the mitochondrial genome of *A. fulloi*.

Feature	Strand	Start sites	Stop sites	Size(bp)	Anticodon	Start codon	End codon	Intergenic nucleotides
trnV	+	1	66	66	UAC			− 2
rrnS	+	65	834	770				5
trnA	+	840	904	65	UGC			− 18
rrnL	+	887	2252	1366				1
trnL1	+	2254	2320	67	UAG			− 3
nad1	+	2318	3265	948		ATA	TAA	9
trnS2	−	3275	3342	68	UGA			2
cytb	−	3345	4481	1137		ATG	TAA	10
nad6	−	4492	5061	570		AAC	TAA	− 2
trnT	−	5060	5127	68	UGU			12
trnP	+	5140	5206	67	UGG			4
nad4l	+	5211	5498	288		ATT	TAA	− 7
nad4	+	5492	6838	1347		ATG	TAA	0
trnH	+	6839	6906	68	GUG			− 3
nad5	+	6904	8581	1678		ATA	T	− 3
trnF	+	8579	8648	70	GAA			16
trnE	−	8665	8732	68	UUC			6
cox1	+	8739	10,280	1542		ATA	TAA	32
trnL2	+	10,313	10,379	67	UAA			0
cox2	+	10,380	11,102	723		ATT	TAA	9
trnK	−	11,112	11,182	71	UUU			13
trnD	+	11,196	11,266	71	GUC			30
atp8	+	11,297	11,458	162		ATT	TAA	− 7
atp6	+	11,452	12,123	672		ATG	TAA	− 1
cox3	+	12,123	12,914	792		ATT	TAA	43
trnG	+	12,958	13,022	65	UCC			18
nad3	+	13,041	13,409	369		ATA	TAG	6
trnS1	−	13,416	13,475	60	UCU			3
trnY	+	13,479	13,549	71	GUA			82
trnN	+	13,632	13,698	67	GUU			129
trnC	−	13,828	13,889	62	GCA			53
trnR	−	13,943	14,007	65	UCG			6
trnQ	−	14,014	14,082	69	UUG			− 2
trnW	−	14,081	14,145	65	UCA			2
nad2	−	14,148	15,164	1017		ATA	TAA	29
trnI	+	15,194	15,263	70	GAU			11
trnM	−	15,275	15,345	71	CAU			0
CR		15,346	15,692	347				

+ represents the heavy strand; − represents the light strand.

Overlapping and intergenic regions are usually detected in hymenopterous mitogenomes^18,20,44^. Generally, the total length of overlapping regions is smaller in size than intergenic regions in most hymenopterous insects^19,45,46^. In the mitogenome of *A. fulloi*, a total of 10 overlapping regions were found, ranging in size from 1 to 18 bp (Table 1). The longest overlapping region was found between trnA and rrnL. The *A. fulloi* mitogenome also contained 24 intergenic spacers with a total length of 531 bp. These intergenic spacers range from 1 to 129 bp (Table 1). The longest gene spacer was located between trnC and trnN. Notably, an overlap between atp6 and atp8 was a common feature of metazoan mitogenomes^47^ and it was also found in *A. fulloi*. Moreover, an overlap between nad4 and nad4l widely occurred among mitogenomes of *A. fulloi* and other hymenopteran parasitoids^17,18,20^, which may be translated as a bicstron^48^.

Significant bias in nucleotide composition is typical in insect mitogenomes^19^ and the nucleotide compositional bias of mitogenomes is usually assessed by non-strand specific (A + T content, G + C content) and strand-specific, namely strand asymmetry (ATskew, GC-skew)^49^. The nucleotides of the *A. fulloi* mitogenome comprise A (39.06%), T (44.77%), C (6.02%), and G (10.15%) (Table 2). The *A. fulloi* mitogenome was highly biased towards A and T nucleotides with an A + T content of 83.83%. In addition, the mitogenome of *A. fulloi* had a negative AT skew (− 0.069) and a positive GC skew (0.255) (Table 2). This indicates that the mitogenome of *A. fulloi* contains more T than A and more G than C, as reported in other mitogenomes of hymenopterous species^20,50^. This common pattern of base composition in mitogenome may be attributed to the highly asymmetric effects of transcription on mutagenesis, including unequal exposure of the strands to DNA damage and the differential chance for repair^51,52^.

**Table 2:** Nucleotide composition of *A. fulloi* mitochondrial genome.

Feature	A%	T%	C%	G%	AT%	GC%	AT Skew	GC Skew
Protein-coding genes	35.13	47.05	8.00	9.82	82.18	17.82	− 0.145	0.102
1st codon position	38.92	38.92	7.92	14.24	77.84	22.16	0.000	0.285
2nd codon position	22.04	52.62	13.71	11.63	74.66	25.34	− 0.410	− 0.082
3rd codon position	44.42	49.63	2.37	3.58	94.05	5.95	− 0.055	0.203
tRNAs	43.08	44.50	4.59	7.83	87.58	12.42	− 0.016	0.261
rRNAs	43.59	43.68	4.03	8.71	87.27	12.74	− 0.001	0.367
CR	41.21	40.92	6.63	11.24	82.13	17.87	0.004	0.258
Whole mitogenome	39.06	44.77	6.02	10.15	83.83	16.17	− 0.069	0.255

### PCGs

**Figure 2:**
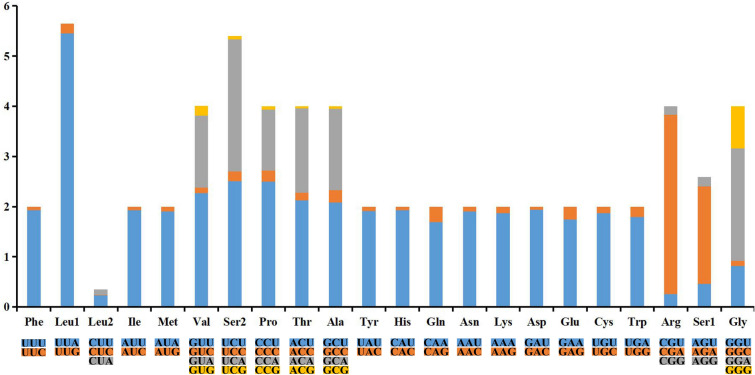
Relative synonymous codon usage of PCGs in the *Anastatus fulloi* mitogenome.

The total length of 13 PCGs was 11,245 bp, accounting for 71.66% of the whole mitogenome. This set of PCGs is conserved in animal mitogenomes, with the exception of nematodes and a bivalve that lack atp8^15^. These PCGs range in size from 162 bp (atp8) to 1678 bp (nad5). The A + T content of all PCGs was 82.18% (Table 2). A remarkably high A + T content (94.05%) was found at the third codon sites of these PCGs, which may partly reflect the high bias towards A and T nucleotides in the mitogenome^19,53,54^. Meanwhile, the AT skew and GC skew of the PCGs were − 0.145 and 0.102, respectively. Of the 13 PCGs, 10 were encoded on the heavy strand, whereas cytb, nad2, and nad6 were encoded on the light strand. In insect mitogenomes, PCGs usually start with ATN codons (ATA, ATT, ATC, and ATG) and terminate with TAA or TAG^55–57^. However, unusual start- and termination codons were simultaneously and exclusively found, such as the start codons of TTG, CGA, GTG, and the incomplete stop codon of T^58–60^ In the *A. fulloi* mitogenome, most PCGs started with ATN, including ATA (nad1-3, nad5, and cox1), ATT (atp8, cox2, cox3, and nad4l) and ATG (atp6 and nad4). However, nad6 used an atypical starting codon of AAC, which has also been found in *Cheirotonus jansoni* and *Prosopocoilus gracilis*^61,62^. All PCGs terminated with TAA except nad3 (TAG) and nad5 (incomplete codon T). Previous studies have inferred that the incomplete termination codon could be completed by posttranscriptional polyadenylation^15,63,64^. The codon usage of PCGs was assessed by the relative synonymous codon usage (RSCU) value (Fig. 2). There is a clear preference for A or T in the third codon. Of the 3736 amino acid-encoding codons, TTA (Leu1), CGA (Arg), TCA (Ser2), and TCT (Ser2) were predominant. Codons such as CGC, AGC, and CTG were not presented.

### tRNAs, rRNAs and the control region

**Figure 3:**
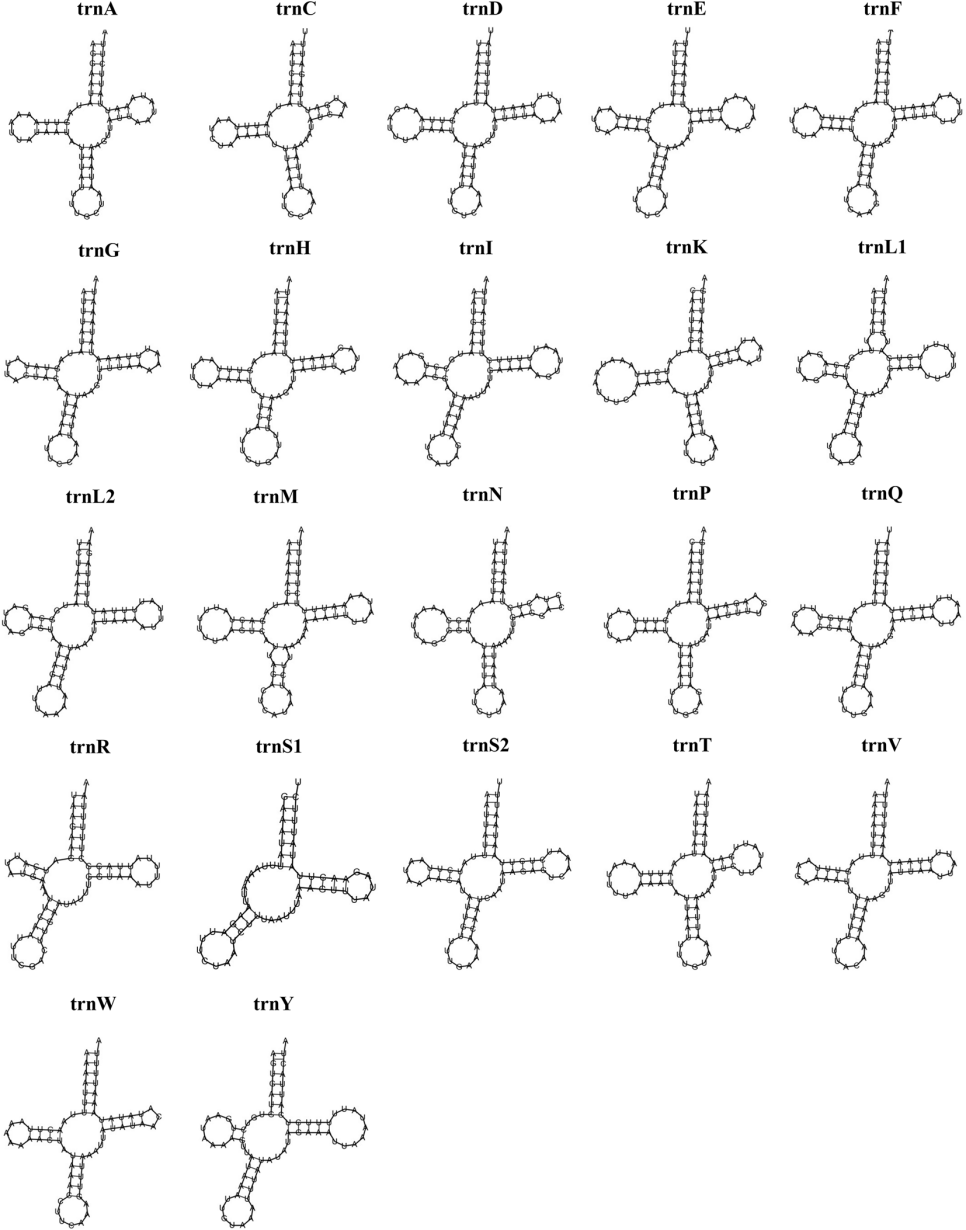
Predicted secondary structures of the 22 typical tRNA genes in the *Anastatus fulloi* mitogenome.

The total length of 22 tRNAs was 1481 bp, accounting for 9.44% of the whole *A. fulloi* mitogenome (Table 2). These tRNAs range from 60 to 71 bp, within the size range observed in hymenopteran parasitoids^17,27,42^ (Table 1). Among these tRNAs, 12 tRNAs were coded in the heavy strand, and the remaining 10 tRNAs were identified in the light strand. These tRNAs had a high A + T content of 87.58%, a slightly negative AT skew value (− 0.016), and a positive GC skew value (0.261). Except for the absence of a dihydrouridine (DHU) arm in trnS1, most tRNAs had a cloverleaf secondary structure (Fig. 3). The loss of the DHU arm in trnS1 is normal in insects^5,65^.

Two rRNAs, i.e., the small ribosomal RNA (rrnS) and large ribosomal RNA (rrnL), were 770 bp and 1366 bp in length, respectively. These lengths are similar to those of most reported hymenopterous insects^27,66^. These rRNAs were located on the heavy strand and were separated by trnA. They consisted of A (43.59%), T (43.68%), C (4.03%), and G (8.71%), with an A + T content of 87.27%. The AT skew and GC skew were − 0.001 and 0.367, respectively.

In insect mitogenomes, the control region, i.e., the A + T-rich region, is associated with replication and transcription^67,68^. This region is variable not only in size but also in base composition^69^. The CR was 3308 bp with an A + T content of 85.9% in *Pteromalus puparum*^70^ but 578 bp with an A + T content of 93.6% in *Spathius agrili*^18^. In addition, repeat structures are usually detected in CR, which is also diverse in both type and size among insect mitogenomes^69^. The high A + T content and presence of repeat regions may result in the failure of obtaining CR sequence due to the inhibition of DNA polymerase^40,71^. Currently, the long and complex control region still presents challenges to obtaining complete mitogenomes in certain groups of parasitoids^19,20,27,41,72,73^. In this study, we failed to obtain the complete segment of the CR by next-generation sequencing or Sanger sequencing. The partial control region obtained for the *A. fulloi* mitogenome was 347 bp with an A + T content of 82.13% and was located between trnM and trnV.

### Gene rearrangement

**Figure 4:**
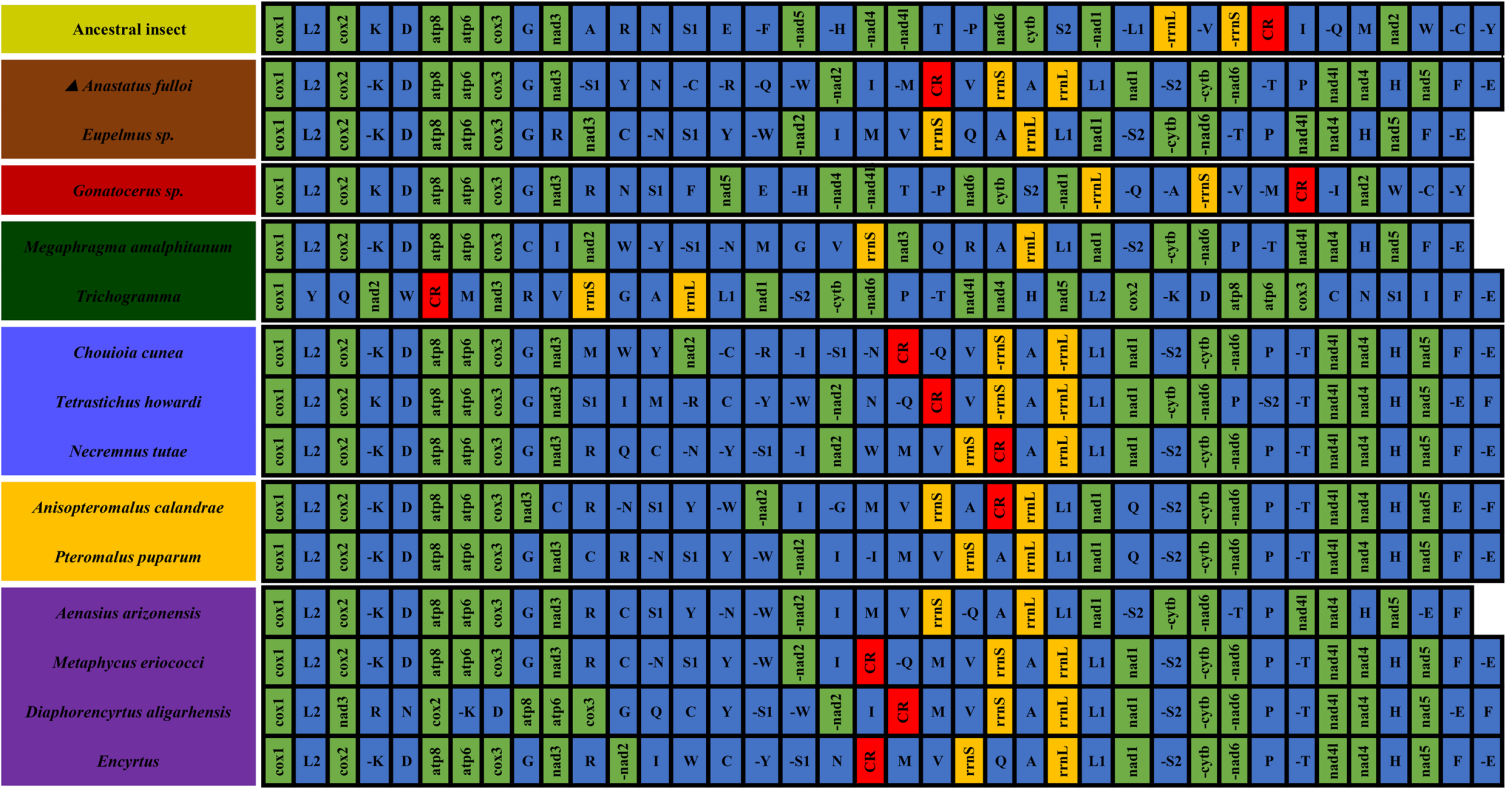
Gene arrangement in the mitogenome of Chalcidoidea.

Mitochondrial gene rearrangements occur frequently in hymenopterous insects^74^, which are important clues to evolution and are regarded as valuable phylogenetic characters for these insects^26^. In this study, a comparison of gene-order data was conducted to examine the gene rearrangements. The gene orders of parasitoid mitogenomes assigned to 6 families are shown in Fig. 4. Species from different genera possessed unique gene orders. Compared with the gene order in the ancestral insect mitogenome, parasitoid mitogenomes exhibit large-scale rearrangement events for tRNA genes and PCGs, as reported in other studies of parasitoid mitogenomes^17,27,75^. In particular, all chalcidoid wasps exhibited an inversion gene block from nad5 to rrnL except *Gonatocerus* sp. Phylogenetically *Gonatocerus* sp. comes closer to the ancestor and agrees with the genetic order as well. Despite another relatively conserved gene block from cox1 to cox3, the remaining genes are highly rearranged. Although the gene orders of these mitogenomes from different families are variable, infrequent rearrangements were found between closely related species. Within Eupelmidae, mitogenomes of *A. fulloi* and *Eupelmus* sp. also exhibit different gene orders that are attributed to the rearrangement of a few tRNAs. Species from the same genus, such as species of *Trichogramma* and or *Encyrtus*, showed the same gene order in mitogenomes. In this study, mitochondrial gene rearrangements are diverse in chalcidoid wasps but occur infrequently in closely related taxa, which might be useful for phylogenetic analysis.

**Figure 5:**
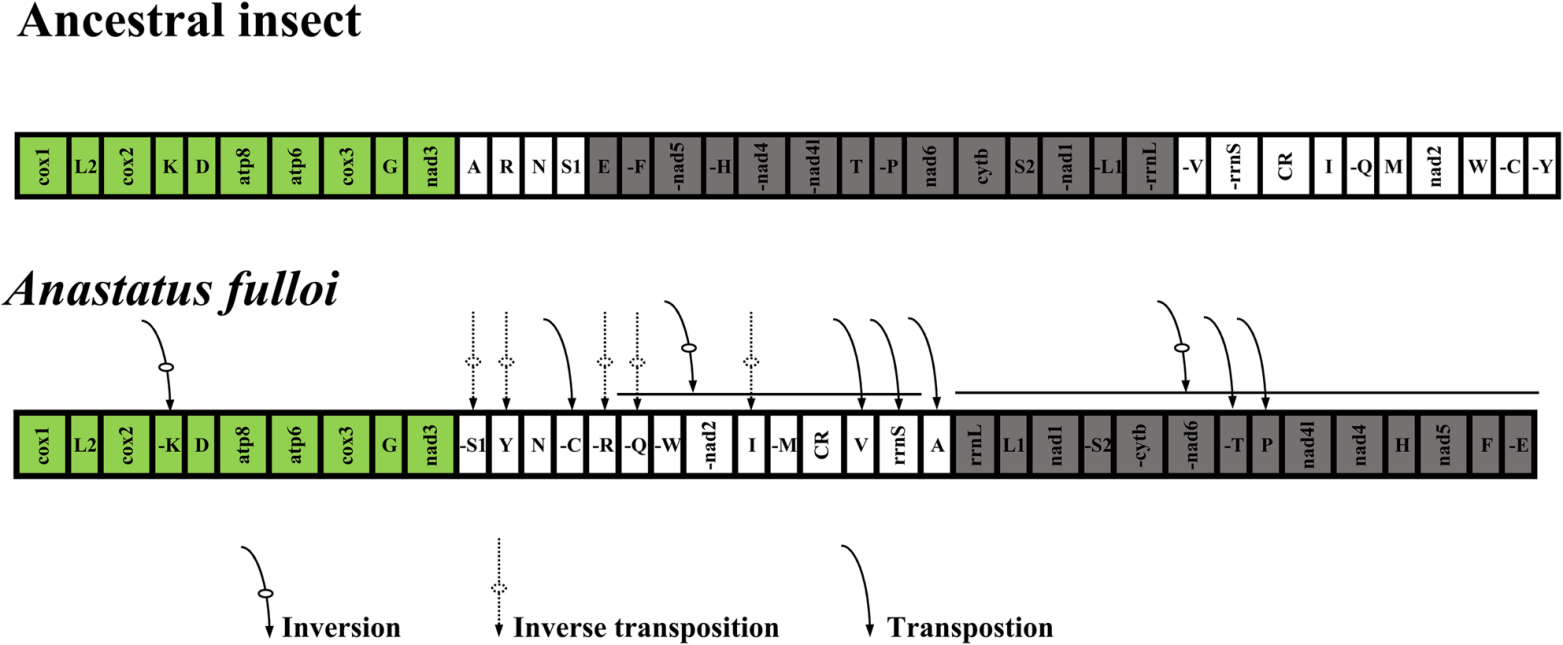
Comparison of gene arrangement between mitogenomes of *Anastatus fulloi* and ancestral insects through CREx analysis. The inverted gene block is shown in grey. Conserved gene block is marked in green.

CREx analysis demonstrated that the gene order of the mitogenome of *A. fulloi* is novel. Compared with the gene order in the ancestral insect mitogenome, the segment between cox1 and nad3 was relatively conserved except for the inversion of trnK, which is the same as *Metaphycus eriococci*, *Encyrtus sasakii*, and *Chouioia cunea*^76^ (Fig. 5). It has been reported that the segment “trnE -trnF -nad5 -trnH -nad4 -nad4l trnT -trnP nad6 cytb” is conserved between *Megraphragma* and *Philotrypesis*^44,77^. However, in the mitogenome of *A. fulloi*, the segment “trnE -trnF -nad5 -trnH -nad4 -nad4l trnT -trnP nad6 cytb trnS2 -nad1 -trnL1 -rrnL” was inversed, and trnT -trnP have exchanged their positions. The segment “-trnV -rrnS CR trnI -trnQ trnM nad2 trnW” was highly rearranged, including the inversions of trnV, rrnS, trnM, nad2 and trnW, position exchanges of trnV and rrnS, trnM and trnI, and the transposition of trnQ (Fig. 5). In addition, trnC and trnY were transported to the segment “trnR trnN trnS1”. The gene order of these tRNA genes was changed to “-trnS1 trnY trnN -trnC -trnR”.

### Phylogenetic analyses

**Figure 6:**
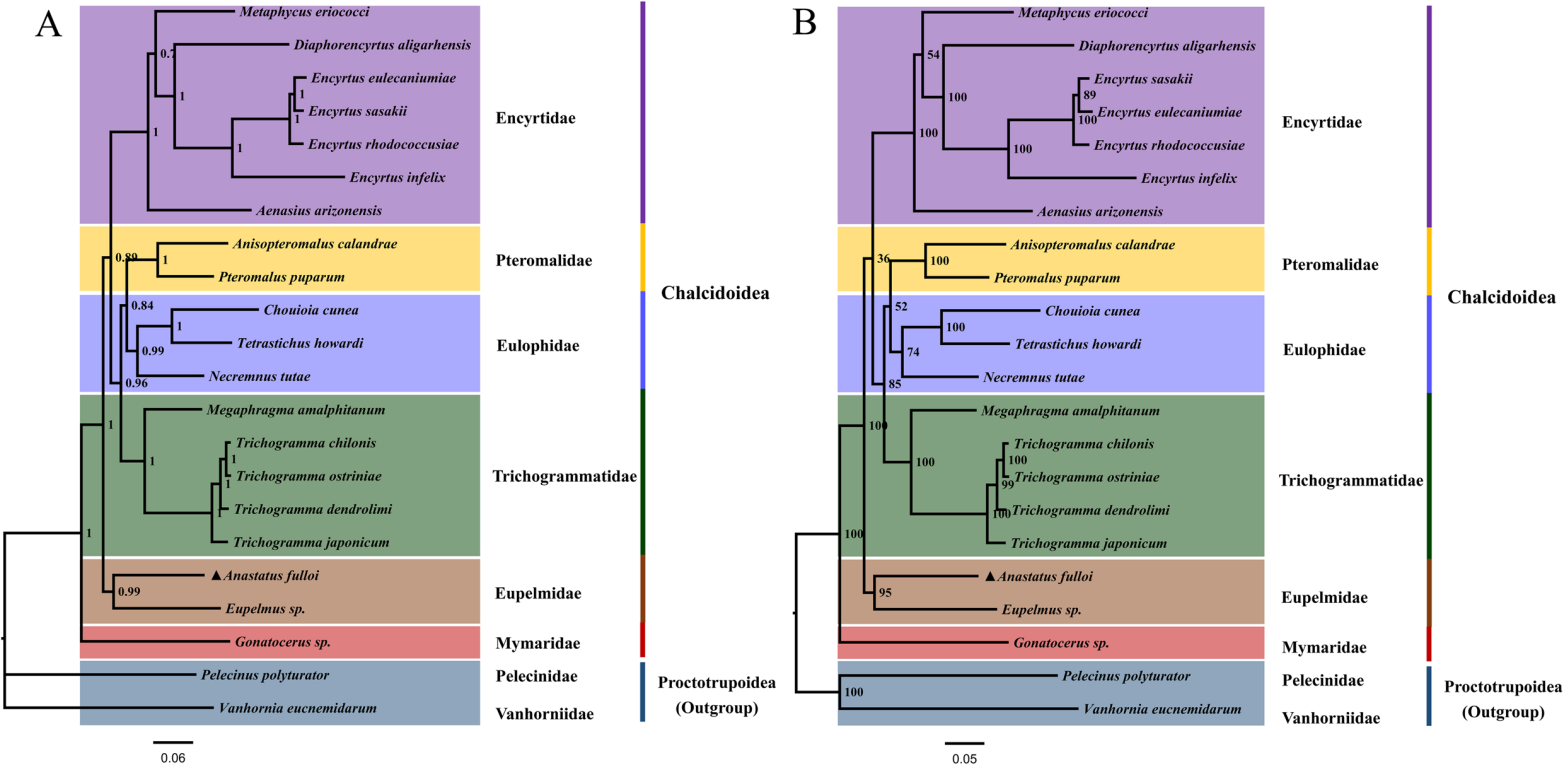
Phylogenetic trees of Chalcidoidea inferred using MrBayes ((**A**) BI) and maximum likelihood ((**B**) ML) analyses based on 13 PCGs.

The phylogenetic relationships of 20 parasitoids within Chalcidoidea were analysed and are displayed in Fig. 6. Maximum likelihood (ML) and Bayesian inference (BI) phylogenetic trees were constructed based on nucleotide sequences of 13 PCGs of these mitogenomes in CIPRES^78^. Although some clades exhibited low support values, the same topological structures were found in two phylogenetic trees. Two species within Eupelmidae, *A. fulloi* and *Eupelmus* sp., were clustered together. Other species within the same families were grouped and separated from other parasitoids in different families. In Chalcidoidea, Eupelmidae is considered to be closely related to Encyrtidae, and to share several of the same features, such as an expanded acropleuron^79,80^. However, this family is not monophyletic, representing a grade rather than a clade^24,81,82^. Some species may show a close relationship to Pteromalidae^79,82^. In the present study, the phylogenetic relationship of parasitoids within Chalcidoidea can be presented as follows: Mymaridae + (Eupelmidae + (Encyrtidae + (Trichogrammatidae + (Pteromalidae + Eulophidae)))). This result is consistent with other reports^27,43^.

## References

[1] Osellame LD, Blacker TS, Duchen MR (2012). Cellular and molecular mechanisms of mitochondrial function. Best Pract. Res. Clin. Endocrinol. Metab..

[2] Bernt M, Braband A, Schierwater B, Stadler PF (2013). Genetic aspects of mitochondrial genome evolution. Mol. Phylogenet. Evol..

[3] Curole JP, Kocher TD (1999). Mitogenomics: Digging deeper with complete mitochondrial genomes. Trends Ecol. Evol..

[4] Shao R, Dowton M, Murrell A, Barker SC (2003). Rates of gene rearrangement and nucleotide substitution are correlated in the mitochondrial genomes of insects. Mol. Biol. Evol..

[5] Cameron SL (2014). Insect mitochondrial genomics: Implications for evolution and phylogeny. Annu. Rev. Entomol..

[6] Lopez-Lopez A, Vogler AP (2017). The mitogenome phylogeny of Adephaga (Coleoptera). Mol. Phylogenet. Evol..

[7] Li Q (2019). The complete mitochondrial genomes of five important medicinal *Ganoderma* species: Features, evolution, and phylogeny. Int. J. Biol. Macromol..

[8] Shang Y (2019). Comparative mitogenomic analysis of forensically important sarcophagid flies (Diptera: Sarcophagidae) and implications of species identification. J. Med. Entomol..

[9] Riyaz M, Shah RA, Savarimuthu I, Kuppusamy S (2021). Comparative mitochondrial genome analysis of *Eudocima salaminia* (Cramer, 1777) (Lepidoptera: Noctuoidea), novel gene rearrangement and phylogenetic relationship within the superfamily Noctuoidea. Mol. Biol. Rep..

[10] Ye F, Li H, Xie Q (2021). Mitochondrial genomes from two specialized subfamilies of Reduviidae (Insecta: Hemiptera) reveal novel gene rearrangements of true Bugs. Genes.

[11] Wang X, Wang J, Dai R (2021). Structural features of the mitogenome of the leafhopper genus *Cladolidia* (Hemiptera: Cicadellidae: Coelidiinae) and phylogenetic implications in Cicadellidae. Ecol. Evol..

[12] Tyagi K (2020). Rearrangement and evolution of mitochondrial genomes in Thysanoptera (Insecta). Sci. Rep..

[13] Dai LS (2018). Mitochondrial genome of *Diaphania indica* (Saunders) (Lepidoptera: Pyraloidea) and implications for its phylogeny. Int. J. Biol. Macromol..

[14] Kumar V (2019). The first complete mitochondrial genome of marigold pest thrips, *Neohydatothrips samayunkur* (Sericothripinae) and comparative analysis. Sci. Rep..

[15] Boore JL (1999). Animal mitochondrial genomes. Nucleic Acids Res..

[16] Du Y, Zhang C, Dietrich CH, Zhang Y, Dai W (2017). Characterization of the complete mitochondrial genomes of *Maiestas dorsalis* and *Japananus hyalinus* (Hemiptera: Cicadellidae) and comparison with other Membracoidea. Sci. Rep..

[17] Chen L (2018). Extensive gene rearrangements in the mitochondrial genomes of two egg parasitoids, *Trichogramma japonicum* and *Trichogramma ostriniae* (Hymenoptera: Chalcidoidea: Trichogrammatidae). Sci. Rep..

[18] Wei S, Tang P, Zheng L, Shi M, Chen X (2010). The complete mitochondrial genome of *Evania appendigaster* (Hymenoptera: Evaniidae) has low A + T content and a long intergenic spacer between atp8 and atp6. Mol. Biol. Rep..

[19] Zhu JC (2018). The first two mitochondrial genomes of the family Aphelinidae with novel gene orders and phylogenetic implications. Int. J. Biol. Macromol..

[20] Powell C, Caleca V, Rhode C, Teixeira DCL, van Asch B (2020). New mitochondrial gene rearrangement in *Psyttalia concolor*, *P. humilis* and *P. lounsburyi* (Hymenoptera: Braconidae), three parasitoid species of economic interest. Insects.

[21] Stahl JM, Babendreier D, Haye T (2019). Life history of *Anastatus bifasciatus,* a potential biological control agent of the brown marmorated stink bug in Europe. Biol. Control.

[22] Yong-Ming C (2020). Performances of six eupelmid egg parasitoids from China on Japanese giant silkworm *Caligula japonica* with different host age regimes. J. Pest Sci..

[23] Peng L, Gibson G, Tang LU, Xiang J (2020). Review of the species of *Anastatus* (Hymenoptera: Eupelmidae) known from China, with description of two new species with brachypterous females. Zootaxa.

[24] Peng LF, Lin NQ (2012). Recent advances in Eupelmidae (Hymenoptera: Chalcidoidea) systematics. Fujian J. Agric. Sci..

[25] Fusu L, Ebrahimi E, Siebold C, Villemant C (2015). Revision of the Eupelmidae Walker, 1833 described by Jean Risbec. Part 1: The slide mounted specimens housed at the Muséum national d’Histoire naturelle in Paris. Zoosystema.

[26] Feng Z (2020). Evolution of tRNA gene rearrangement in the mitochondrial genome of ichneumonoid wasps (Hymenoptera: Ichneumonoidea). Int. J. Biol. Macromol..

[27] Wu Y (2020). Novel gene rearrangement in the mitochondrial genome of *Pachyneuron aphidis* (Hymenoptera: Pteromalidae). Int. J. Biol. Macromol..

[28] Bankevich A (2012). SPAdes: A new genome assembly algorithm and its applications to single-cell sequencing. J. Comput. Biol..

[29] Coil D, Jospin G, Darling AE (2015). A5-Miseq: An updated pipeline to assemble microbial genomes from Illumina MiSeq data. Bioinformatics.

[30] Kurtz S (2004). Versatile and open software for comparing large genomes. Genome Biol..

[31] Walker BJ (2014). Pilon: An integrated tool for comprehensive microbial variant detection and genome assembly improvement. PLoS ONE.

[32] Bernt M (2007). CREx: Inferring genomic rearrangements based on common intervals. Bioinformatics.

[33] Zhang D (2020). PhyloSuite: An integrated and scalable desktop platform for streamlined molecular sequence data management and evolutionary phylogenetics studies. Mol. Ecol. Resour..

[34] Katoh K, Standley DM (2013). MAFFT multiple sequence alignment software version 7: Improvements in performance and usability. Mol. Biol. Evol..

[35] Ranwez V, Douzery E, Cambon C, Chantret N, Delsuc F (2018). MACSE v2: Toolkit for the alignment of coding sequences accounting for frameshifts and stop codons. Mol. Biol. Evol..

[36] Castresana J (2000). Selection of conserved blocks from multiple alignments for their use in phylogenetic analysis. Mol. Biol. Evol..

[37] Kalyaanamoorthy S, Minh BQ, Wong T, von Haeseler A, Jermiin LS (2017). ModelFinder: Fast model selection for accurate phylogenetic estimates. Nat. Methods.

[38] Dowton M, Cameron SL, Austin AD, Whiting MF (2009). Phylogenetic approaches for the analysis of mitochondrial genome sequence data in the Hymenoptera—A lineage with both rapidly and slowly evolving mitochondrial genomes. Mol. Phylogenet. Evol..

[39] Korkmaz EM, Aydemir HB, Temel B, Budak M, Başıbüyük HH (2017). Mitogenome evolution in Cephini (Hymenoptera: Cephidae): Evidence for parallel adaptive evolution. Biochem. Syst. Ecol..

[40] Wei SJ, Li Q, van Achterberg K, Chen XX (2014). Two mitochondrial genomes from the families Bethylidae and Mutillidae: Independent rearrangement of protein-coding genes and higher-level phylogeny of the Hymenoptera. Mol. Phylogenet. Evol..

[41] Shen ZC, Chen L, Chen L, Li YX (2019). Information from the mitochondrial genomes of two egg parasitoids, *Gonatocerus* sp. and *Telenomus* sp., reveals a controversial phylogenetic relationship between Mymaridae and Scelionidae. Genomics.

[42] Yang J, Liu HX, Li YX, Wei ZM (2019). The rearranged mitochondrial genome of *Podagrion* sp. (Hymenoptera: Torymidae), a parasitoid wasp of mantis. Genomics.

[43] Xing ZP (2021). Complete mitochondrial genome of a parasitoid, *Trichogramma chilonis* (Hymenoptera: Chalcidoidea: Trichogrammatidae) and phylogenetic analysis. Mitochondrial DNA B Resour..

[44] Xiao JH, Jia JG, Murphy RW, Huang DW (2011). Rapid evolution of the mitochondrial genome in Chalcidoid wasps (Hymenoptera: Chalcidoidea) driven by parasitic lifestyles. PLoS ONE.

[45] Wei SJ, Shi M, He JH, Sharkey M, Chen XX (2009). The complete mitochondrial genome of *Diadegma semiclausum* (Hymenoptera: Ichneumonidae) indicates extensive independent evolutionary events. Genome.

[46] Zhang QH, Huang P, Chen B, Li TJ (2018). The complete mitochondrial genome of *Orancistrocerus aterrimus aterrimus* and comparative analysis in the family Vespidae (Hymenoptera, Vespidae, Eumeninae). ZooKeys.

[47] Campbell NJ, Barker SC (1999). The novel mitochondrial gene arrangement of the cattle tick, *Boophilus microplus*: Fivefold tandem repetition of a coding region. Mol. Biol. Evol..

[48] Stewart JB, Beckenbach AT (2005). Insect mitochondrial genomics: The complete mitochondrial genome sequence of the meadow spittlebug *Philaenus spumarius* (Hemiptera: Auchenorrhyncha: Cercopoidae). Genome.

[49] Negrisolo E, Babbucci M, Patarnello T (2011). The mitochondrial genome of the ascalaphid owlfly *Libelloides macaronius* and comparative evolutionary mitochondriomics of neuropterid Insects. BMC Genomics.

[50] Wu QL (2014). The complete mitochondrial genome of *Taeniogonalos taihorina* (Bischoff) (Hymenoptera: Trigonalyidae) reveals a novel gene rearrangement pattern in the Hymenoptera. Gene.

[51] Francino MP, Ochman H (1997). Strand asymmetries in DNA evolution. Trends Genet..

[52] Hassanin A, Leger N, Deutsch J (2005). Evidence for multiple reversals of asymmetric mutational constraints during the evolution of the mitochondrial genome of Metazoa, and consequences for phylogenetic inferences. Syst. Biol..

[53] Chai HN, Du YZ (2012). The complete mitochondrial genome of the pink stem borer, *Sesamia inferens*, in comparison with four other Noctuid moths. Int. J. Mol. Sci..

[54] Ma Z (2015). Comparative mitogenomics of the genus *Odontobutis* (Perciformes: Gobioidei: Odontobutidae) revealed conserved gene rearrangement and high sequence variations. Int. J. Mol. Sci..

[55] Yi J, Que S, Xin T, Xia B, Zou Z (2016). Complete mitochondrial genome of *Thitarodes pui* (Lepidoptera: Hepialidae). Mitochondrial DNA A DNA Mapp. Seq. Anal..

[56] Li J (2018). Mitochondrial genome characteristics of two Sphingidae insects (*Psilogramma increta* and *Macroglossum stellatarum*) and implications for their phylogeny. Int. J. Biol. Macromol..

[57] Wang W (2019). Characterization of the complete mitochondrial genomes of two species of the genus *Aphaena Guerin-Meneville* (Hemiptera: Fulgoridae) and its phylogenetic implications. Int. J. Biol. Macromol..

[58] Wang JJ, Yang MF, Dai RH, Li H, Wang XY (2018). Characterization and phylogenetic implications of the complete mitochondrial genome of Idiocerinae (Hemiptera: Cicadellidae). Int. J. Biol. Macromol..

[59] Huang Y (2019). Comparative mitochondrial genome analysis of *Grammodes geometrica* and other noctuid insects reveals conserved mitochondrial genome organization and phylogeny. Int. J. Biol. Macromol..

[60] Sun Z (2019). Mitochondrial genome of *Phalantus geniculatus* (Hemiptera: Reduviidae): trnT duplication and phylogenetic implications. Int. J. Biol. Macromol..

[61] Shao LL (2014). Complete mitochondrial genome sequence of *Cheirotonus jansoni* (Coleoptera: Scarabaeidae). Genet. Mol. Res..

[62] Wu YY, Cao YY, Fang J, Wan X (2016). The first complete mitochondrial genome of stag beetle from China, *Prosopocoilus gracilis* (Coleoptera, Lucanidae). Mitochondrial DNA A DNA Mapp. Seq. Anal..

[63] Anderson S (1981). Sequence and organization of the human mitochondrial genome. Nature.

[64] Ojala D, Montoya J, Attardi G (1981). tRNA punctuation model of RNA processing in human mitochondria. Nature.

[65] Juhling F (2012). Improved systematic tRNA gene annotation allows new insights into the evolution of mitochondrial tRNA structures and into the mechanisms of mitochondrial genome rearrangements. Nucleic Acids Res..

[66] Aydemir MN, Korkmaz EM (2020). Comparative mitogenomics of Hymenoptera reveals evolutionary differences in structure and composition. Int. J. Biol. Macromol..

[67] Taanman JW (1999). The mitochondrial genome: Structure, transcription, translation and replication. Biochim. Biophys. Acta..

[68] Cameron SL (2008). Mitochondrial genome organization and phylogeny of two vespid wasps. Genome.

[69] Zhang D, Hewitt GM (1997). Insect mitochondrial control region: A review of its structure, evolution and usefulness in evolutionary studies. Biochem. Syst. Ecol..

[70] Yan Z (2019). Mitochondrial DNA and their nuclear copies in the parasitic wasp *Pteromalus puparum*: A comparative analysis in Chalcidoidea. Int. J. Biol. Macromol..

[71] Hu M, Jex AR, Campbell BE, Gasser RB (2007). Long PCR amplification of the entire mitochondrial genome from individual helminths for direct sequencing. Nat. Protoc..

[72] Mao M, Valerio A, Austin AD, Dowton M, Johnson NF (2012). The first mitochondrial genome for the wasp superfamily Platygastroidea: The egg parasitoid *Trissolcus basalis*. Genome.

[73] Oliveira DS, Gomes TM, Loreto EL (2016). The rearranged mitochondrial genome of *Leptopilina boulardi* (Hymenoptera: Figitidae), a parasitoid wasp of *Drosophila*. Genet. Mol. Biol..

[74] Dowton M, Cameron SL, Dowavic JI, Austin AD, Whiting MF (2009). Characterization of 67 mitochondrial tRNA gene rearrangements in the Hymenoptera suggests that mitochondrial tRNA gene position is selectively neutral. Mol. Biol. Evol..

[75] Lin ZJ (2021). Comparative analysis reveals the expansion of mitochondrial DNA control region containing unusually high G-C tandem repeat arrays in *Nasonia vitripennis*. Int. J. Biol. Macromol..

[76] Tang X (2021). The mitochondrial genome of a parasitic wasp, *Chouioia cunea* Yang (Hymenoptera: Chalcidoidea: Eulophidae) and phylogenetic analysis. Mitochondrial DNA B Resour..

[77] Nedoluzhko AV (2016). Mitochondrial genome of *Megaphragma amalphitanum* (Hymenoptera: Trichogrammatidae).. Mitochondrial DNA A DNA Mapp. Seq. Anal..

[78] Miller MA (2015). A RESTful API for access to phylogenetic tools via the CIPRES science gateway. Evol. Bioinform..

[79] LaSalle J (1987). New world Tanaostigmatidae (Hymenoptera, Chalcidoidea). Contrib. Am. Entomol. Inst..

[80] Munro JB (2011). A molecular phylogeny of the Chalcidoidea (Hymenoptera). PLoS ONE.

[81] Gary APG (1989). Phylogeny and classification of Eupelmidae, with a revision of the world genera of Calosotinae and Metapelmatinae (Hymenoptera: Chalcidoidea). Mem. Entomol. Soc. Can..

[82] Gibson GA (2008). Description of *Leptoomus janzeni*, N. Gen. and N. sp. (Hymenoptera: Chalcidoidea) from Baltic amber, and discussion of its relationships and classification relative to Eupelmidae, Tanaostigmatidae and Encyrtidae. Zootaxa.

